# Optical Sensor for Real-Time Detection of Trichlorofluoromethane

**DOI:** 10.3390/s19030632

**Published:** 2019-02-02

**Authors:** Maiko Girschikofsky, Dimitrij Ryvlin, Siegfried R. Waldvogel, Ralf Hellmann

**Affiliations:** 1 Applied Laser and Photonics Group, University of Applied Sciences Aschaffenburg, 63743 Aschaffenburg, Germany; ralf.hellmann@h-ab.de; 2 Institute of Organic Chemistry, Johannes Gutenberg University Mainz, 55128 Mainz, Germany; waldvogel@uni-mainz.de

**Keywords:** Bragg grating, sensor, supramolecular chemistry, cyclodextrin, trichloroflouromethane

## Abstract

Trichlorofluoromethane was once a promising and versatile applicable chlorofluorocarbon. Unaware of its ozone-depleting character, for a long time it was globally applied as propellant and refrigerant and thus led to significant thinning of the ozone layer and contributed to the formation of the so-called ozone hole. Although production and application of this substance were gradually reduced at an early stage, we still face the consequences of its former careless use. Today, trichlorofluoromethane is released during recycling processes of waste cooling devices, traded on the black market, and according to recent findings still illegally manufactured. Here, we present an optical sensor device for real-time in-situ detection and measurement of this environmentally harmful chlorofluorocarbon. The described sensor is based on a planar Bragg grating that is functionalized with cyclodextrin derivatives and operates on the principle of a chemical sensor. In our study, the sensor is sensitized using *per*-methyl-, *per*-ethyl-, and *per*-allyl-substituted α-, β-, and γ-cyclodextrins as affinity materials for airborne trichlorofluoromethane. These functional coatings have been proven to be highly efficient, as an up to 400-times stronger signal deflection could be achieved compared to an identical but uncoated sensor. The presented sensor device shows instantaneous response to trichlorofluoromethane exposure, and features a limit-of-detection of less than 25 ppm, depending on the applied affinity material.

## 1. Introduction

In the middle of the 20th century, technically manufactured chlorofluorocarbons (CFCs) were frequently used as refrigerants, propellants, and solvents. The ozone-depleting effect of these substances, however, only became known in the early 1970s [1,2] and was only taken seriously by the discovery of an abnormally low stratospheric ozone concentration near the South Pole in 1985 [3] which was later metaphorically referred to as the ozone hole. Soon after, following the Montreal Protocol in 1987, the international community decided to phase-out production and use of these and likewise ozone-depleting substances (ODS) [4]. And in fact, as a result of the agreement, a turnaround and steady decline in measured CFC concentration could be observed from the mid-1990s [5,6]. However, the gradual reduction of CFC production and consumption was only set to be completed in the years 1996 and 2010 for developed and developing countries, respectively. Until the end of these grace periods, considerable quantities of ODS were accumulated in terms of either stockpiled or product-bound CFCs [7]. Through the ongoing illegal trade of feedstock chlorofluorocarbons [8,9] and temporal as well as deliberately conducted destruction of CFC-containing products [10], even today, a noteworthy amount of these severely ODS still leaks into the atmosphere and damages the ozone layer [7]. A recent study by Montzka et al. from 2018 actually states an alarming re-increase in the global emission of trichlorofluoromethane (referred to as CFC-11; also known as Freon-11, or R-11), one of the most abundant CFCs that still contributes more than 20% of all chlorine reaching the upper atmosphere [11,12]. Since CFC-11 is chemically almost inert, it represented an ideal blowing agent for fabrication of polyurethane foams that were applied in packaging, furniture and as thermal insulating material in cooling equipment such as refrigerators and freezers [13]. Although very little of the CFC-11 blowing agent is released during the lifetime of these products, the fracturing of such foams, e.g., in the shredding of waste cooling devices, causes a release of the blowing agent, whose detection and extraction still represents a challenge for modern recycling plants [14,15].

The ongoing illegal trade of trichlorofluoromethane, its release during recycling processes, and the latest findings on the illegal resumption of CFC-11 production demonstrate the critical nature of the situation and clearly underlines that even after three decades following the Montreal Protocol the challenge of cleaning up the atmosphere of CFCs is still topical and a serious concern. Consequently, the sufficient detection and measurement of this environmentally hazardous pollutant are necessary to take appropriate actions. Today’s state-of-the-art technologies for the detection or measurement of airborne CFC-11 are typically based on chromatography [16,17], spectroscopy [18,19], semiconductor technology [20,21], or visualized chemical reactions [22,23]. These technologies, however, do not allow a selective and online in-situ CFC-11 measurement in real time.

Here we present a chemo-optical sensor system for the detection and measurement of airborne trichlorofluoromethane. The sensor system is based on optical planar Bragg gratings functionalized by means of cyclodextrin derivative affinity materials and thus allows a selective and highly sensitive in-situ online measurement of CFC-11.

## 2. Planar Bragg Grating Sensor

The here presented sensor approach is based on waveguide Bragg grating technology [24]. In principle, Bragg gratings (BGs) represent a periodic modulation of the refractive index within an optical waveguide, such as optical fibers or planar waveguides. These optical elements feature the peculiarity to act as a dielectric band-stop for a specific wavelength according to the Bragg grating condition (1).

(1) $$m\;\lambda_{B}=2\;n_{\mathrm{eff}}\;\Lambda$$

**Figure 1:**
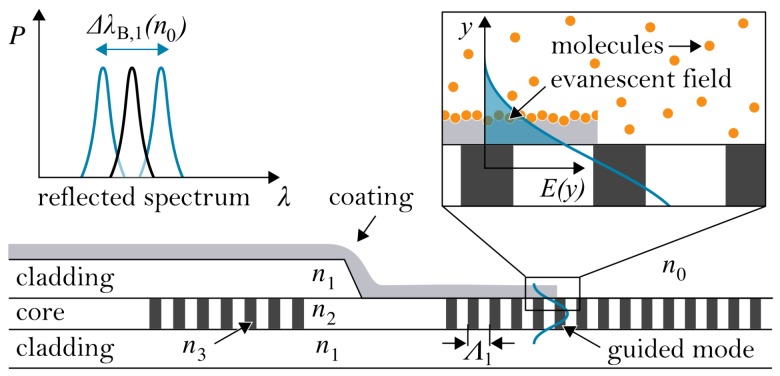
Cross-section of the planar Bragg grating showing two BGs located in the waveguide core of which one is uncovered by the upper cladding. The close-up depicts the evanescent field interaction of the guided mode with the surrounding medium and the principle of surface functionalization. The graph shows the reflected Bragg wavelength’s dependency on the surrounding medium’s refractive index.

This suppressed and thus reflected wavelength $\lambda_{B}$, which is referred to as the Bragg wavelength, is thereby depending on the effective refractive index $n_{\mathrm{eff}}$ of the guided mode, the period of the refractive index modulation Λ and the order of reflection *m* (which is a natural number) [25,26]. Since the reflected Bragg wavelength is depending on $n_{\mathrm{eff}}$ and Λ, any variable that influences one or both parameters leads variation of the reflected Bragg wavelength and can accordingly be detected and monitored with appropriate interrogation equipment [27]. The most prominent examples of variables that are well detectable with BGs are temperature and deformation, as demonstrated by the authors and others elsewhere [28,29,30]. A further area of application can be exploited as soon as the guided light in the waveguide can interact with the ambient medium. By omitting the cladding at the Bragg grating, a fraction of the guided mode, the so-called evanescent field, can penetrate into the surrounding medium, whose refractive index therefore becomes part of $n_{\mathrm{eff}}$ and by that means influences the reflected Bragg wavelength (Figure 1). Hence, any change of the surrounding refractive index leads to a detectable and traceable sensor response that can be monitored with appropriate interrogation equipment.

The major advantages of this optical sensor technology are its lightweight design, strong electromagnetic anti-jamming quality, chemical as well as thermal resistance, signal-multiplexing and considerably low attenuation which allows remote monitoring of multiple sensors simultaneously that can be placed even in correspondingly challenging environments [25,27,31,32].

The applied planar Bragg grating (PBG) sensor is based on a multilayer structure of silica (SiO${}_{2}$) on a silicon wafer [33,34]. During the epitaxial growth of SiO${}_{2}$ by flame hydrolysis, intermediately, germanium (Ge) is added to the process resulting in a stack-structure of SiO${}_{2}$, Ge-doped SiO${}_{2}$ and SiO${}_{2}$ on top of the wafer. The doping results in photo-sensibility of the middle layer, which allows simultaneous inscription of both, waveguide core and Bragg grating using a direct writing technique based on UV-laser refractive index modification [35]. By this measure, waveguide BGs with different grating periods are inscribed into the middle layer, vertically enclosed by the SiO${}_{2}$ cladding and horizontally enclosed by non-modified Ge-doped SiO${}_{2}$ cladding. Each Bragg grating leads to a defined Bragg reflection peak according to the Bragg condition (1). To guarantee evanescent field interaction of the guided mode with the surrounding medium, the silica cladding above two BGs is removed by wet etching resulting in an ellipse-shaped sensing window. For further enhancement of refractive index sensitivity, the sensor surface is covered by a 50 nm thin titan dioxide layer which causes a slight offset of the guided mode into the direction of surface and hence a more pronounced evanescent field interaction with the surrounding medium. Two remaining BGs, located between sensing window and waveguide coupling facet, remain buried under the top-cladding, are thus uninfluenced by the surrounding medium and serve as reference for intrinsically interfering influences such as temperature changes. A detailed description of the PBG fabrication process, the sensor’s characteristics as well as its applicability can be found by the authors and others elsewhere [33,34,36]. For its application, a single mode optical fiber (SMF) pig-tail that comprises an FC/APC connector is butt-coupled and bonded to the coupling facet of the PBG-sensor using a UV-curable adhesive. The thus ready-to-use sensor chip is connected to a static optical sensing interrogator (SIS: Lab II by Stratophase comprising sm125 by micron optics) that operates in telecom wavelength range at a resolution of Δλ = 1 pm and allows detection and tracking of the reflected Bragg wavelengths at a sampling rate of $f_{s}$ = 2 Hz.

## 3. Substituted Cyclodextrins for Sensor Sensitization

**Figure 2:**
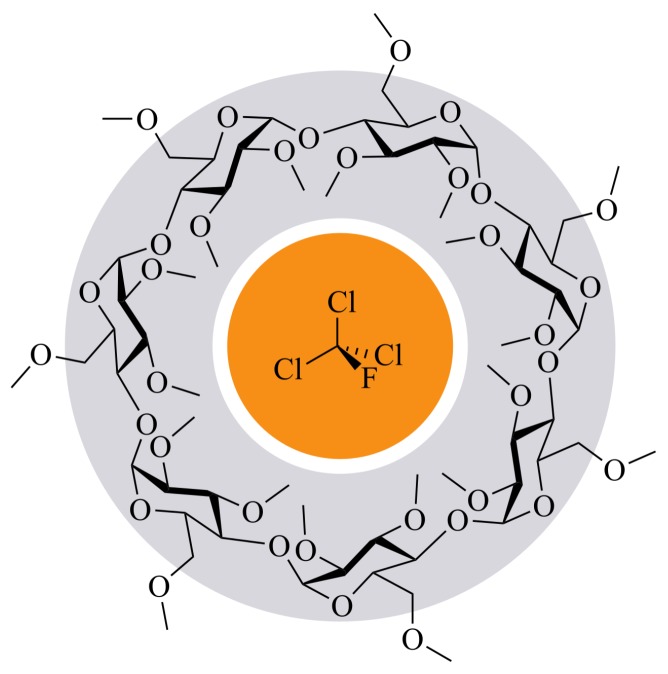
Illustration of the inclusion complex formation of trichlorofluoromethane (inner orange circle) in heptakis(2,3,6-tri-*O*-methyl)-β-cyclodextrin (outer gray ring).

For a detection of the ozone-depleting trichlorofluoromethane, the PBG-sensor must be sensitized by an appropriate affinity material to show an analyzable response at an exposure to this highly volatile pollutant. A promising substance class of affinity materials for sufficient sensitization of PBG-sensors are cyclodextrins (CyD) [37]. The principle behind the sensitizing characteristic of this class is based on molecular recognition, which is represented by non-covalent supramolecular inclusion complex formation (ICF) of the CFC-11 guest molecule within the CyD hosts (Figure 2). By application of CyDs on the sensitive area of the PBG-sensor, continuous host-guest interactions occur which increase the retention time and thus the analyte concentration on the sensor surface.

Generally, cyclodextrin is a cyclic oligosaccharide typically containing six, seven or eight glucopyranose units linked by an α-1,4 glycosidic bond and is referred to as α-, β- and γ-cyclodextrin, respectively [38,39,40]. The interior of the toroid-shaped structures is hydrophobic while the exterior is hydrophilic. Therefore, cyclodextrin can form non-covalent but solid host-guest complexes with hydrophobic molecules of suitable dimension [38,40,41,42]. Other characteristics such as viscosity, solubility, and ICF-selectivity can moreover be modified by sufficient substitution of the CyD’s hydroxyl groups [38,39,43]. In the here presented approach, the following nine *per*-substituted cyclodextrin derivatives were synthesized and applied as affinity materials to the PBG-sensor:

*Per*-methyl substituted cyclodextrin derivatives:
  CyD1: hexakis(2,3,6-tri-*O*-methyl)-α-cyclodextrin
  CyD2: heptakis(2,3,6-tri-*O*-methyl)-β-cyclodextrin
  CyD3: octakis(2,3,6-tri-*O*-methyl)-γ-cyclodextrin
*Per*-ethyl substituted cyclodextrin derivatives:
  CyD4: hexakis(2,3,6-tri-*O*-ethyl)-α-cyclodextrin
  CyD5: heptakis(2,3,6-tri-*O*-ethyl)-β-cyclodextrin
  CyD6: octakis(2,3,6-tri-*O*-ethyl)-γ-cyclodextrin
*Per*-allyl substituted cyclodextrin derivatives:
  CyD7: hexakis(2,3,6-tri-*O*-allyl)-α-cyclodextrin
  CyD8: heptakis(2,3,6-tri-*O*-allyl)-β-cyclodextrin
  CyD9: octakis(2,3,6-tri-*O*-allyl)-γ-cyclodextrin

**Figure 3:**
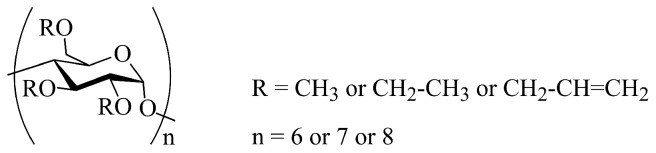
Short hand formula of the applied *per*-substituted cyclodextrin derivatives.

The respective short hand formula of the applied cyclodextrin derivatives is depicted in Figure 3. The cyclodextrin derivatives CyD1-CyD6 were synthesized according to reaction conditions described in [44,45], whereas CyD7-CyD9 were synthesized following routes described in [46,47]. Structure and purity of each synthesized cyclodextrin derivative is confirmed by NMR-spectroscopy (AVANCE II 400 by Bruker Corporation, Billerica, MA, USA) and mass spectrometry (6545 Q-ToF by Agilent Technologies, Santa Clara, CA, USA).

The application of the cyclodextrin derivatives as sensitization layer on the PBG-sensor’s surface is performed by dip-coating technology (WPTL5-0.01 by MTI Corporation, Richmond, CA, USA). For the coating process, the CyD derivatives are dissolved in tetrahydrofuran (THF) resulting in concentrations of 50 mg·mL${}^{-1}$. During the coating process, the PBG-sensor is vertically immersed into the individual CyD/THF solution at an immersion speed of 200 mm·min${}^{-1}$ and a dwell time of approx. 3 s. The withdrawal speed, responsible for the later layer thickness, is chosen individually for the respective CyD derivatives. For the derivatives CyD1, CyD2, and CyD3, a withdrawal speed of 150 mm·min${}^{-1}$, 100 mm·min${}^{-1}$, and 80 mm·min${}^{-1}$, respectively, results in a layer thickness of 140 ± 10 nm. As for the derivatives CyD4, CyD5, and CyD6, a withdrawal speed of 130 mm·min${}^{-1}$, 100 mm·min${}^{-1}$, and 120 mm·min${}^{-1}$, respectively, leads to a layer thickness of 135 ± 10 nm. And for the derivatives CyD7, CyD8, and CyD9 a withdrawal speed of 100 mm·min${}^{-1}$, 60 mm·min${}^{-1}$, and 75 mm·min${}^{-1}$, respectively, creates a layer thickness of 85 ± 10 nm. Although higher thickness of the sensitization layer provides higher sensitivity of the PBG-sensor [37], it also leads to a pronounced decoupling of the guided light resulting in a reduction or even loss of the sensor signal. Taking into account these controversial aspects, the thicknesses of the respective sensitization layers are individually chosen by empirical determination. Therefore, during the coating procedure, the reflected Bragg wavelengths are simultaneously monitored to ensure sufficient sensitization. The layer thickness of the applied CyD derivatives is determined by stylus profilometry (DektakXT by Bruker Corporation, Billerica, MA, USA).

## 4. Experimental Setup

**Figure 4:**
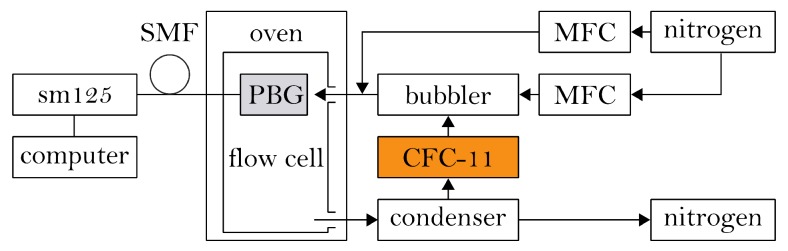
Schematic illustration of the experimental setup depicting the static optical sensing interrogator, the temperature-controlled gas-flow chamber containing the sensor, and the gas mixing unit.

A schematic illustration of the experimental setup is depicted in Figure 4. For the investigation of the sensitized PBG-sensor’s response to airborne CFC-11 exposure, a gas mixing unit is applied that is based on the principle of vapor pressure saturation according to the ISO 6145-9:2009 standard [48].

Here, a nitrogen carrier gas flow (with a purity of 99.998%) is divided into two separate lines, each controlled by a mass-flow-controller (MFC; 5050S by Brooks Instrument, Hatfield, PA, USA). The first line is led through a temperature-controlled bubbler, filled with trichlorofluoromethane and is thus fully saturated with CFC-11. The concentration of the analyte in the nitrogen carrier gas flow is thereby only based on the analyte’s temperature and the system pressure. In the experimental setup, the bubbler temperature is set to 15 ${}^{\circ }$C at an ambient pressure of approx. 1 bar, leading to a CFC-11 concentration of 74.1 vol% for the fully saturated nitrogen flow [49]. Subsequent to enrichment, the analyte saturated gas flow is mixed with the pure nitrogen gas flow of the second line. Therefore, by adjusting the analyte’s temperature and volumetric flow of the two gas lines, a defined concentration of the analyte in the recombined gas flow is achieved. This gas flow of defined analyte concentration is then directed over the PBG-sensor, which is placed in a temperature-controlled gas-flow cell and connected to the described source and detector interrogation system. The temperature of this cell is set to 35 ${}^{\circ }$C to prevent condensation of the analyte at the sensor surface, which might cause falsification of the sensor signal. After passing the gas-flow cell, the CFC-11 enriched nitrogen gas flow is directed through a condenser which is tempered to −78 ${}^{\circ }$C using a dry-ice/acetone cooling bath enabling a separation and recollection of the CFC-11 from the nitrogen gas flow.

Measurements are performed with an uncoated PBG-sensor and the sensor being functionalized with the different cyclodextrin derivatives. After installing the sensors in the gas-flow cell and prior to the measurements, the sensors are conditioned for approx. 24 h at 35 ${}^{\circ }$C and a constant nitrogen flow of 200 mL·min${}^{-1}$. During the measurements, a continuously increasing CFC-11 concentration in the range between 1% and 10% with intermediate purging by pure nitrogen is applied to the sensor surface, while simultaneously monitoring the reflected wavelengths of the BGs. The measurements are performed at ambient pressure, a sensor temperature of 35 ${}^{\circ }$C, an analyte temperature of 15 ${}^{\circ }$C and a gas-flow rate of 200 mL·min${}^{-1}$. The observed sensor signal deflections are tracked and correlated with the respectively applied trichlorofluoromethane concentration.

## 5. Results and Discussion

**Figure 5:**
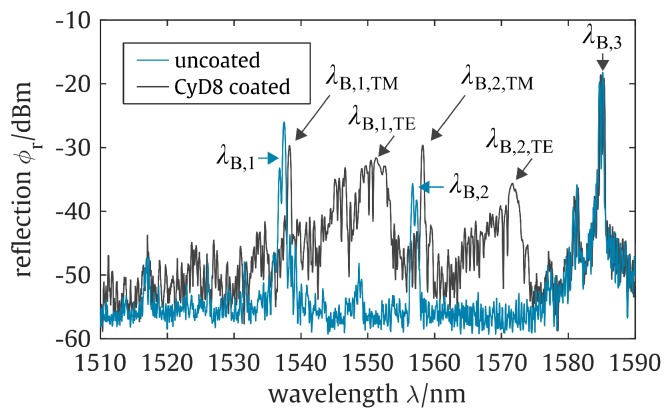
Reflection spectra of an uncoated planar Bragg grating sensor and the sensor coated with cyclodextrin derivative CyD8.

Figure 5 depicts the reflected spectra of the PBG-sensor prior to and after the dip-coating procedure. While the uncoated PBG-sensor features three distinct Bragg reflection peaks $\lambda_{B,1}$, $\lambda_{B,2}$, and $\lambda_{B,3}$, the sensor coated with one of the cyclodextrin derivatives distinguishes five Bragg reflection peaks.

**Figure 6:**
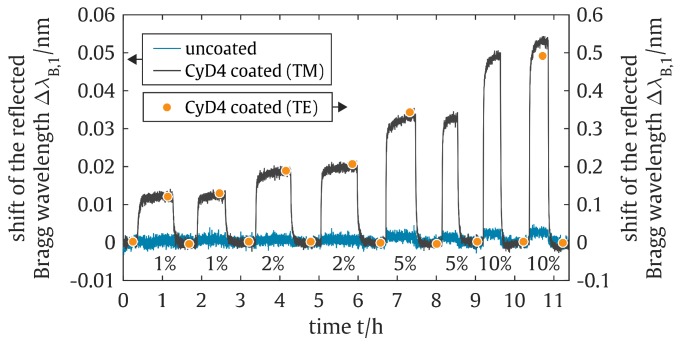
Shift of the reflected Bragg wavelengths for an uncoated PBG-sensor and the sensor coated with CyD4 to an increasing CFC-11 concentration. The discreetly recorded Bragg wavelength shift of the TE-mode reflection refers to the secondary axis.

Here, $\lambda_{B,3}$ remains unchanged by the functionalization as this particular reflection peak can be dedicated to a reference Bragg grating located beneath the top-cladding and allows an identification of intrinsically interfering influences, such as temperature changes [33,50]. The Bragg reflection peaks $\lambda_{B,1}$ and $\lambda_{B,2}$ can be dedicated to two BGs that are in the ellipse-shaped sensing window that features no silica top-cladding. As a result of functionalization, these two reflection peaks were found to firstly obtain a wavelength shift towards higher wavelengths and secondly to split up into two Bragg reflection peaks each. The wavelength shifts can be dedicated to a noticeable refractive index increase of the sensor’s surrounding medium due to the replacement of air with the functionalization layer. As a consequence of this refractive index increase and due to the evanescent field interaction of the guided mode with the surrounding medium, $n_{\mathrm{eff}}$ increases as well and thus $\lambda_{B}$ (see condition (1)). The peak splitting, on the other hand, can be dedicated to birefringence characteristics of the waveguide structure [33]. While $\lambda_{B,1}$, $\lambda_{B,2}$, and $\lambda_{B,3}$ already indicate this effect by featuring a double-peak-like shape, whereby the individual spikes can be assigned to the propagating mode’s TE- and TM-polarization that experience slightly different effective refractive indices, the functionalization significantly enhances this effect. Here, the respective reflection peaks of the propagating mode’s TE-polarization $\lambda_{B,n,\mathrm{TE}}$ are found to obtain a significantly more pronounced Bragg wavelength shift as compared to the reflection peaks of the propagating mode’s TM-polarization $\lambda_{B,n,\mathrm{TM}}$. This disparity can be dedicated to a more distinct evanescent field overlap of the guided TE-mode with the applied functionalization layer as compared to the TM-mode. Mostly undesired, here, the birefringent effect can be well exploited for the sensor application. As with the sensor’s response to the coating procedure, the TE-polarization caused sensor signals also feature a significantly higher sensitivity to refractive index changes of the sensitization layer. Due to the ICF of CFC-11 guest molecules with the cyclodextrin hosts, the refractive index of the sensitization layer, which interacts with the evanescent field of the guided mode, is altered and following condition (1), leads to a shift of the reflected Bragg wavelengths. Figure 6 depicts the corresponding PBG-sensor signal deflection due to an increasing trichlorofluoromethane concentration for an uncoated sensor and a sensor coated with the cyclodextrin derivative CyD5.

Each sensitized PBG-sensor shows an immediate and reproducible response to the applied CFC-11 concentration as expressed in a sharp signal rise (a so-called red-shift) that merges into saturation. The intermediately performed purge with pure nitrogen results in an equally erratic signal decrease (a so-called blue-shift) which returns to the signal’s initial state and, thus, highlights the reversibility of the sensor system. The TE-polarization caused sensor signals are found to accomplish an approx. ten-times higher signal deflection to analyte exposure as compared to the TM-polarization caused sensor signals. However, the more indistinct characteristic of the TE-polarization caused reflection peaks combined with the applied peak detection algorithm led to complications in monitoring the respective wavelength shifts. This could be overcome by performing discrete records of the reflection spectra and a subsequent application of a more sufficient peak detection algorithm [51]. As compared to a non-functionalized PBG-sensor, the cyclodextrin sensitized Bragg grating sensor is found to exhibit an up to 400-fold wavelength shift (TE-polarization caused signal of the CyD2-functionalized sensor) and accordingly proves the excellent suitability of the cyclodextrin derivatives as affinity materials for the chemical sensing of CFC-11.

**Figure 7:**
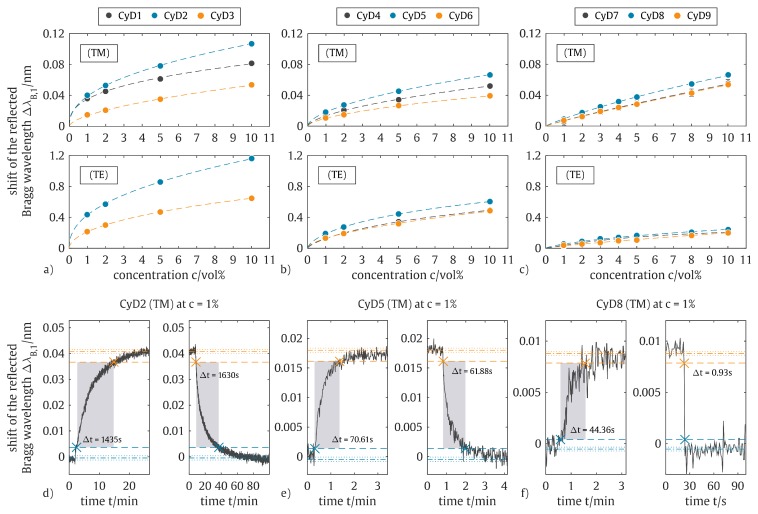
Sensor signal equilibria of the sensitized PBG-sensor depending on the applied CFC-11 concentration comprising Langmuir-Freundlich isotherm fits for the sensor coated with (**a**) *per*-methyl substituted CyD derivatives, (**b**) *per*-ethyl substituted CyD derivatives, and (**c**) *per*-allyl substituted CyD derivatives. Sensor signal’s temporal response to 1 vol% CFC-11 exposure and pure nitrogen purge for the sensor coated with (**d**) a *per*-methyl substituted CyD derivative, (**e**) a *per*-ethyl substituted CyD derivative, and (**f**) a *per*-allyl substituted CyD derivative.

In general, the red-shift of the reflected Bragg wavelength at CFC-11 exposure and the blue-shift at nitrogen purging can be dedicated to an ICF driven refractive index modification of the sensitization layer, where the cyclodextrin molecules assimilating a CFC-11 molecule apparently feature a higher refractive index. The signal’s merging into a state of saturation, which can be found at higher wavelengths for higher trichlorofluoromethane concentrations, is dedicated to a dynamic equilibrium of complex formation and guest release which shifts, depending on the number of available guest molecules [39]. The binding strength of those ICFs depends among others on how well the inclusion complex fits together [39]. Therefore, the analysis of the sensor’s sensitivity based on sensitization layers of different cyclodextrin derivatives is of interest and provides the fabrication of a highly selective sensor matrix as demonstrated by the authors elsewhere [52,53,54]. Consequently, to investigate the sensor’s sensitivity based on the sensitization layers of different cyclodextrin derivatives, the sensor signal’s equilibria are analyzed as a function of the applied CFC-11 concentration (Figure 7a–c).

For all investigated affinity materials, the β-CyD derivatives show the highest signal deflection as compared to the equally substituted α-CyD and γ-CyD derivatives. With respect to the applied substitution group, the PBG-sensor sensitized with *per*-methyl substituted cyclodextrin exhibits the most pronounced response, while the sensor sensitized with *per*-ethyl and *per*-allyl substituted cyclodextrin features lesser signal deflections. It is noticeable that regardless of the applied affinity material, the PBG-sensor signals show a non-linear response to the applied CFC-11 concentration. This can be attributed to isothermal variation of CFC-11 guest molecule adsorption at the sensitization layer in dependence on the analyte concentration, which can be described by a combined Langmuir-Freundlich isotherm [55], written as:

(2) $$B=\frac{N_{t}\;a\;F^{m}}{1+a\;F^{m}}$$

where *B* and *F* are the concentrations of bound and free guests, respectively, $N_{t}$ the total number of binding sites, *a* being related to the median binding affinity constant $K_{0}$ = $a^{1/m}$ and *m* the heterogeneity index that varies from 0 (heterogeneous material) to 1 (homogeneous material). At its limits, Equation (2) furthermore reduces to either the Langmuir or Freundlich isotherm. Consequently, the relation is capable of modeling both, homogeneous as well as heterogeneous binding surfaces [55] and agrees well with the investigated sensor responses by featuring a coefficient of determination of R${}^{2}$ ≥ 0.99 for each fit. According to the determined isotherm relations and considering the spectral resolution of the applied sensing interrogator, the PBG-sensor provides a limit-of-detection (LOD) as depicted in Table 1. The determined LODs consider a signal deflection of $\Delta \lambda_{B}$ = 10 pm, which is more than three times the signal’s background noise σ ≈ 3 pm.

The PBG-sensor’s response to an exposure of CFC-11 and subsequently pure nitrogen (Figure 7d–f) depicts a significant difference in the required time to reach equilibrium condition of the signal. Here, the PBG-sensor functionalized with *per*-allyl substituted α, β, or γ cyclodextrin exhibits a faster response than the sensor coated with the respective *per*-ethyl substituted cyclodextrin, while both feature a considerably faster response as compared to the sensor sensitized with the respective *per*-methyl substituted cyclodextrin. In other words, the signal’s response times decrease with increasing length of the affinity material’s substitution group. The authors assume that the *per*-methyl substituted cyclodextrin affinity materials form an adsorbent layer that allows diffusion mechanisms of the adsorbate while the *per*-allyl substituted cyclodextrin functionalizations result in a more homogeneous and denser layer. This hypothesis is supported by the signal’s determined isotherm relations of the PBG-sensor functionalized with *per*-allyl substituted cyclodextrin derivatives, which exhibit a heterogeneity index close to 1 of the Langmuir-Freundlich relation. The heterogeneity indices for the isotherm relations of the PBG-sensor functionalized with *per*-methyl and *per*-ethyl substituted cyclodextrin derivatives are found to be around 0.4 and 0.6, respectively. Furthermore, it is noticeable that a slower complex formation yields a higher sensitivity of the sensor. These features indicate a more stable inclusion complex of the *per*-methyl substituted cyclodextrin derivatives with CFC-11 as compared to the other investigated cyclodextrin affinity materials and are in excellent agreement with our previous findings on the ICF of CFC-11 with hexakis(2,3,6-tri-*O*-methyl)-α-cyclodextrin (CyD1) [45], where even a spontaneous crystallization of the inclusion complex at higher concentrations could be observed.

## 6. Conclusions

In this contribution, we demonstrated the application of a robust and lightweight optical PBG sensor system functionalized with non-toxic *per*-methyl, *per*-ethyl, and *per*-allyl substituted α-, β-, and γ-cyclodextrins for a real-time in-situ detection and measurement of the environmentally harmful propellant trichlorofluoromethane. The sensor fabrication, its easy sensitization with the cyclodextrin derivatives, and the experimental setup were described and the sensor’s application for CFC-11 measurements together with the resulting data have been demonstrated and discussed.

Functionalizing the PBG-sensor with the cyclodextrin derivatives results in an up to 400-fold signal increase to CFC-11 exposure (TE-polarization caused signal of the CyD2-functionalized sensor) as compared to a non-functionalized sensor. Each sensitized PBG-sensor shows an immediate and reproducible response as expressed in a sharp signal rise that merges into saturation, whose level is found to follow a Langmuir-Freundlich isotherm relation. Depending on the applied cyclodextrin affinity material, saturation can be reached in less than 60 s and a LOD of less than 25 ppm can be achieved, which represents a performance comparable to state-of-the-art semiconductor-based CFC-sensor devices [20,21].

The easy fabrication, its fast response and high sensitivity qualifies the presented opto-chemical sensor system for the detection of CFC-11 and paves the way for the development of a highly selective sensor array that allows a reliable detection and measurement of airborne trichlorofluoromethane.

## Figures and Tables

**Table 1:** Estimated CFC-11 limit-of-detection of the PBG-sensor sensitized with the different cyclodextrin derivatives for the TM- and TE-polarization caused Bragg reflections as well as the sensor response at a concentration of 1 vol%.

Functionalization	LOD		Response to 1 vol%	
	(TM)	(TE)	Rise Time	Fall Time
CyD1	130 ppm	—	3092 s	3341 s
CyD2	320 ppm	5 ppm	1435 s	1630 s
CyD3	4300 ppm	25 ppm	1473 s	1866 s
CyD4	4500 ppm	220 ppm	339 s	429 s
CyD5	2600 ppm	75 ppm	71 s	62 s
CyD6	7200 ppm	85 ppm	143 s	168 s
CyD7	11,300 ppm	1700 ppm	24 s	291 s
CyD8	8300 ppm	750 ppm	45 s	1 s
CyD9	11,600 ppm	2000 ppm	350 s	14 s
